# The serotonergic psychedelic N,N-dipropyltryptamine alters information-processing dynamics in in vitro cortical neural circuits

**DOI:** 10.1162/netn_a_00408

**Published:** 2024-12-10

**Authors:** Thomas F. Varley, Daniel Havert, Leandro Fosque, Abolfazl Alipour, Naruepon Weerawongphrom, Hiroki Naganobori, Lily O’Shea, Maria Pope, John Beggs

**Affiliations:** School of Informatics, Computing, and Engineering, Indiana University, Bloomington, IN, USA; Department of Psychological and Brain Sciences, Indiana University, Bloomington, IN, USA; Vermont Complex Systems Center, University of Vermont, Burlington, VT, USA; Department of Physics, Indiana University, Bloomington, IN, USA; Program in Neuroscience, Indiana University, Bloomington, IN, USA; Indiana University, Bloomington, IN, USA

**Keywords:** Psychedelics, Information theory, Synergy, Partial information decomposition, Network neuroscience, Transfer entropy, Computation, Information dynamics

## Abstract

Most of the recent work in psychedelic neuroscience has been done using noninvasive neuroimaging, with data recorded from the brains of adult volunteers under the influence of a variety of drugs. While these data provide holistic insights into the effects of psychedelics on whole-brain dynamics, the effects of psychedelics on the mesoscale dynamics of neuronal circuits remain much less explored. Here, we report the effects of the serotonergic psychedelic N,N-diproptyltryptamine (DPT) on information-processing dynamics in a sample of in vitro organotypic cultures of cortical tissue from postnatal rats. Three hours of spontaneous activity were recorded: an hour of predrug control, an hour of exposure to 10-*μ*M DPT solution, and a final hour of washout, once again under control conditions. We found that DPT reversibly alters information dynamics in multiple ways: First, the DPT condition was associated with a higher entropy of spontaneous firing activity and reduced the amount of time information was stored in individual neurons. Second, DPT also reduced the reversibility of neural activity, increasing the entropy produced and suggesting a drive away from equilibrium. Third, DPT altered the structure of neuronal circuits, decreasing the overall information flow coming into each neuron, but increasing the number of weak connections, creating a dynamic that combines elements of integration and disintegration. Finally, DPT decreased the higher order statistical synergy present in sets of three neurons. Collectively, these results paint a complex picture of how psychedelics regulate information processing in mesoscale neuronal networks in cortical tissue. Implications for existing hypotheses of psychedelic action, such as the entropic brain hypothesis, are discussed.

## Author Summary

In the last two decades, there has been an explosion of interest in the neural substrates of the psychedelic experience. Almost all of this work has focused on human neuroimaging with modalities like fMRI, EEG, MEG, and so forth. These approaches provide a coarse, whole-brain perspective on psychedelic drug action but miss the fine-scale changes in neuron-level firing. This study uses in vitro recordings of organotypic cultures to explore how the serotonergic psychedelic N,N-diproptyltryptamine alters information-processing dynamics in networks of a few hundreds neurons. We find robust alterations to information dynamics, including changes to the global connectivity patterns suggestive of a changing integration/segregation balance. These results should inform future work on the neurobiological basis of psychedelic drug actions.

## INTRODUCTION

Serotonergic psychedelics such as lysergic acid diethylamide (LSD), psilocybin, and mescaline are known to induce intense, exotic states of consciousness that depart markedly from normal day-to-day patterns of cognition and perception (Nichols, 2016). Since the turn of the century, there has been a resurgence of interest in the scientific exploration of psychedelic states, with a particular focus on using whole-brain neuroimaging technologies to understand the neural correlates of the psychedelic experience. In typical recent studies, adult human volunteers are given a psychedelic, and then brain activity is recorded for analysis, which can then be compared with self-reported phenomonological experiences (such as the experience of ego dissolution; Lebedev et al., 2015; Letheby & Gerrans, 2017; Stoliker, Egan, Friston, & Razi, 2022; Tagliazucchi et al., 2016) or clinical presentations (such as depression; Carhart-Harris et al., 2017; Kuburi et al., 2022; or PTSD; Singleton et al., 2023). Human neuroimaging studies have been done using almost every available modality, including fMRI (for a review of existing fMRI dataset, see McCulloch et al., 2022), EEG (for a partial review of EEG studies, see Tófoli & de Araujo, 2016), and magnetoencephalography (MEG) (Carhart-Harris et al., 2016). Collectively, these studies have painted a complex picture of the effects of different psychedelics on whole-brain, macroscale activity, with one of the most-discussed effects being a general increase in the entropy (or “complexity”) of macroscale brain activity (for review, see Sarasso et al., 2021; although, for a recent study into which specific measures of entropy replicate, see McCulloch et al., 2023). This apparent link prompted Carhart-Harris and colleagues to propose the so-called “entropic brain hypothesis” (EBH), which posits a link between the information density of spontaneous brain activity and the perceptual richness or lability of conscious experience (Carhart-Harris, 2018; Carhart-Harris et al., 2014).

There have been far fewer attempts to understand the microscale effects of psychedelics at the level of circuits of cortical neurons. This creates something of a schism in the field of psychedelic science: At the level of individual neurons, ligands, and receptors, the pharmacological properties of psychedelics are well understood (Nichols, 2016), and at the level of the entire brain, the effects of psychedelics on brain dynamics are beginning to crystallize as well (increased complexity of spontaneous activity, etc.; Carhart-Harris, 2018; Sarasso et al., 2021). However, the intermediary dynamics, composed of networks of interacting neurons induced by psychedelics at the “mesoscale,” which presumably form the causal substrate of the high-level dynamical changes, remains largely unexplored.

The few studies that have been done in this space have largely focused on single measures, such as firing rate (Rajpal & Guerrero, 2023; Wood, Kim, & Moghaddam, 2012) or coherence (Brys et al., 2022). Our goal with this study was a more comprehensive analysis of how a serotonergic psychedelic alters the information-processing dynamics of neural circuits. Information dynamics (J. T. Lizier, 2013) is a branch of information theory concerned with the understanding how distributed systems “compute” their trajectories through configuration space over time. Prior work has shown that the information dynamics framework applied to spiking neural activity is powerful enough to reveal meaningful differences in the cognitive state and behavior in awake, behaving animals (Varley, Sporns, Schaffelhofer, Scherberger, & Dann, 2023) and has been used to explore the structure and dynamics of organotypic cultures (Faber, Timme, Beggs, & Newman, 2019; Ito et al., 2011, 2014; Kajiwara et al., 2021; Newman, Varley, Parakkattu, Sherrill, & Beggs, 2022; Timme, Marshall, et al., 2016). Here, following Varley et al. (2023), we applied the information dynamics framework to spontaneous spiking activity collected from organotypic cultures before, during, and after exposure to the serotonergic psychedelic N,N-dipropyltryptamine (DPT), with the aim of creating a comprehensive portrait of the way that the psychedelic drug alters information dynamics at the circuit level.

Organotypic cultures are a commonly used in the in vitro model system for exploring the spiking dynamics of neuronal networks (Beggs & Plenz, 2003). For details, see the Materials and Methods section, but briefly, slices of neural tissue are cultured from the brains of approximately 1-week-old rat pups postmortem, and after a period of incubation, the spiking activity of individual neurons can be directly recorded with a multielectrode array. While these in vitro have their limitations (e.g., there is no behavior to associate changes in brain activity with), as simplified model systems, they provide a high degree of access and control to cortical activity that are usually inaccessible (Humpel, 2015).

DPT is a serotonergic psychedelic of the tryptamine class and a close analog of the more well-known psychedelic, N,N-dimethyltryptamine (DMT; one of the active ingredients in Ayahuasca). DPT has been known to science since the early days of psychedelic research: As early as 1962, it was being explored as a tryptamine analog of psilocybin (Faillace, Vourlekis, & Szara, 1967; Szara, Hearst, & Putney, 1962). By the 1970s, it had become an object of clinical research, being tested as a treatment for alcoholism (Grof, Soskin, Richards, & Kurland, 1973) and later to test if its mystical experience-producing properties might be of use for terminal cancer patients facing the end of their lives (Richards, Rhead, Dileo, Yensen, & Kurland, 1977). In the years following the passing of the Controlled Substances Act, scientific and clinical interest in DPT waned; however, it was never criminalized in the United States and it remains unscheduled at the Federal level. Despite its legality, DPT remains much less well-known among the general public than its more famous siblings such as psilocybin, DMT, mescaline, and LSD. A notable exception to this is its use by a religious organization based in New York City, The Temple of the True Inner Light, which uses DPT as a religious sacrament of the *Temple of the True Inner Light* (n.d.). Despite its somewhat unusual history and status, pharmacological research has shown it to be a standard serotonergic psychedelic of the tryptamine class, with activity mediated by both the 5-HT_2A_ and 5-HT_1A_ receptors, which is typical of the class of drugs in question (Fantegrossi et al., 2008; Li, Rice, & France, 2007). Its legal status, and close relationship to more well-known, scheduled drugs, made it an excellent compound for this study.

## RESULTS

### Summary of Methods

**Figure 1.**
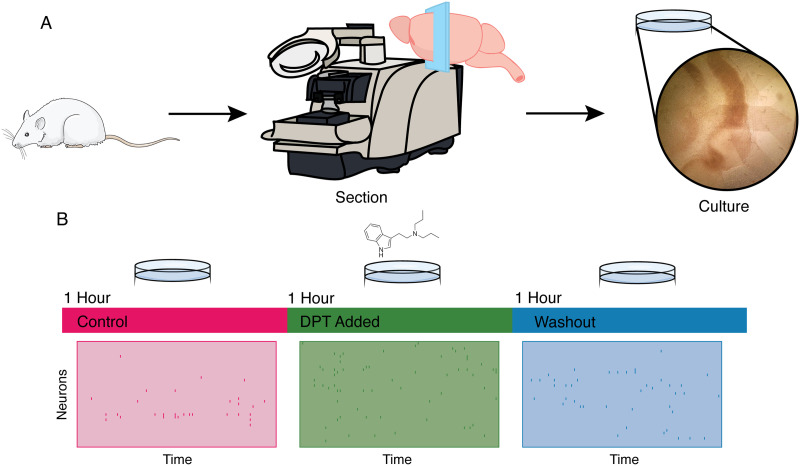
Visual explanation of methods. (A) The slices are prepared from the cortical tissue of Sprague-Dawley rats, sectioned, and cultured in vitro for a period of 2 weeks. (B) Following incubation, cultures were recorded for 3 hr: 1 hr before drug administration in a control medium (pink), 1 hr while being exposed to a 10-*μ*M solution of N,N-DPT (pink), and finally, for 1 hr under control conditions after washout (blue). Example raster plots showing spikes for each condition are showed.

Here, we will briefly outline the methods and analyses presented in this paper. For visualization, see Figure 1. For more details, see the Materials and Methods section. To investigate how DPT affects neuronal activity at the mesoscale of cortical neuronal networks, we chose to use organotypic cultures of rat somatosensory cortex. Organotypic cultures preserve some of the layered structure that is typical of cortex, yet are compact and easily accessible for fluid changes, as needed in this study. Moreover, these cultures have been shown to display many of the emergent properties reported from recordings of in vivo systems, including wavelike structures (Sanchez-Vives & McCormick, 2000), synchrony (Cappaaert, Lopes da Silva, & Wadman, 2009), gamma oscillations (Fisahn, Pike, Buhl, & Paulsen, 1998), repeating activity patterns (Rolston, Wagenaar, & Potter, 2007), and neuronal avalanches (Beggs & Plenz, 2003). They also display a rich club structure of effective connectivity (Ito et al., 2014), as reported in many other neural systems (Dann, Michaels, Schaffelhofer, & Scherberger, 2016; van den Heuvel, Kahn, Goñi, & Sporns, 2012; Varley et al., 2023). Following prior work (Ito et al., 2011; Tang et al., 2008), organotypic cultures of cortical tissue were taken from 5-day postnatal rats, and after a 2-week incubation period, spontaneous spiking activity was recorded on a 512-electrode array. Recordings lasted for a 3-hr period; in the first hour, the cultures were recorded in their standard environment of cell media. In the second hour, the cultures were exposed to a 10-*μ*M solution of N,N-DPT at a perfusion rate of 3 ml/min. In the third hour, the drug was washed out, and a subsequent hour of control condition was recorded. We then analyzed how the statistics of population firing activity varied between control, drug, and washout recordings.

The data were spike-sorted using the kilosort3 package (Pachitariu, Sridhar, & Stringer, 2023), and analyzed using the *information dynamics* framework (J. T. Lizier, 2013) with the aid of the IDTxl package. Information dynamics uses the mathematics of information theory to describe the statistical structure of temporally extended processes, with the ultimate goal of creating an effective model of the distributed “computations” the system is performing. Due to variability between cultures (such as which specific regions of the somatosensory cortex the initial culture was taken from, precise placement of the electrode array, etc.), we aggregated all neurons into a single sample for analysis and do not explore culture-level differences.

The various information-dynamic measures can be grouped into three general categories: first-order measures that describe the dynamics of individual neurons. We considered the Shannon entropy of the spike train (a measure of activity intensity), the active information storage (AIS; J. T. Lizier, Prokopenko, & Zomaya, 2012; M. Wibral, Lizier, Vögler, Priesemann, & Galuske, 2014) (a measure of temporal autocorrelation), and the entropy production (Lynn, Cornblath, Papadopoulos, Bertolero, & Bassett, 2021; Roldán & Parrondo, 2012) (a measure of how time-reversible the dynamics of the elements are). The second set of measures were second order, describing the interactions between pairs of elements. We considered the multivariate transfer entropy (Bossomaier, Barnett, Harré, & Lizier, 2016; J. Lizier & Rubinov, 2012; Novelli, Wollstadt, Mediano, Wibral, & Lizier, 2019), a measure of information flow from a “source” neuron to a “target” neuron, and for each culture, inferred a multivariate transfer entropy network after (Varley et al., 2023). In addition to the amount of information flow between neurons in bits, we also characterized the local topology of the directed networks with the local clustering coefficient (Watts & Strogatz, 1998). The final set of “higher order” measures was the statistical synergy between pairs of sources onto a single target (for review, see Newman et al., 2022). This serves as a measure of information modification (J. T. Lizier, Flecker, & Williams, 2013) or nontrivial “computation” in circuits of multiple interacting neurons (N. M. Timme, Ito, et al., 2016). Since almost all of the measures returned values spanning multiple orders of magnitude (a typical feature of neural data; Buzsáki & Mizuseki, 2014), we log-transformed the values for statistical analysis. Furthermore, since not every neuron was active in every condition, we filtered the neurons and only included those cells that were active in all three conditions; this ensures the validity of the repeated-measures design. Finally, information-theoretic measures (active information storage, multivariate transfer entropy, synergy) were normalized as described in Newman et al. (2022) by dividing the measure by the target entropy, which accounts for the variable firing rates that could confound the data. For visualization of the information-theoretic measures, see Figure 3.

Collectively, this suite of measures presents a multidimensional perspective on how the serotonergic psychedelic N,N-DPT alters computational dynamics in cortical neuronal circuits. We have defined various technical and reference terms throughout this paper (see the Discussion section), and all the measures are detailed more formally in the Materials and Methods section.

### First-Order Measures

Friedman’s *χ*^2^ found a significant difference in the log-transformed Shannon entropy (*Q* ≈ 174.89, *p* ≈ 1.06 × 10^−38^). Post hoc analysis found that the DPT condition had significantly higher log-transformed entropy (−2.29 ± 0.72) than both the control condition (−2.57 ± 0.99, *t* ≈ −12.92, *p* ≈ 2.06 × 10^−36^, Cohen’s *d* = −0.32), and the washout condition (−2.58 ± 0.9, *t* ≈ 16.88, *p* ≈ 6.07 × 10^−59^, Cohen’s *d* = 0.35), but there was no significant difference between the control and washout conditions. This is consistent with whole-brain level findings that serotonergic psychedelics increase the overall entropy of brain activity (Carhart-Harris et al., 2014; Sarasso et al., 2021). When considering the log-transformed entropy production (a measure of irreversibility) of the spike trains, Friedman’s test found a significant difference between conditions (*Q* ≈ 80.42, *p* ≈ 3.44 × 10^−18^), and post hoc analysis once again found a small, but significantly higher entropy production (greater irreversibility) in the DPT condition (−4.13 ± 1.39) when compared with the control condition (−4.47 ± 1.57, *t* ≈ −8.29, *p* ≈ 3.71 × 10^−16^, Cohen’s *d* = −0.23) and the washout condition (−4.46 ± 1.45, *t* ≈ 10.22, *p* ≈ 2.29 × 10^−23^, Cohen’s *d* = 0.23), but not between control and washout. Recent work on human neuroimaging has found that loss of consciousness is associated with increased reversibly of brain activity (de la Fuente et al., 2023; G-Guzmán et al., 2023), and so the finding that a psychedelic like DPT is associated with an increase in entropy production suggests that time reversibility may be a more general marker of conscious states.

Interestingly, we found no significant differences in the log-transformed AIS between any of the conditions; however, we did find strong, significant differences in the maximum search depth for the embedding lag (*Q* ≈ 348.17, *p* ≈ 2.49 × 10^−76^). The maximum search depth can be understood as the “time-horizon” of the neuron’s memory: the maximum distance into the past that still contains information about the immediate future. Post hoc analysis found that all three conditions were distinct. The DPT condition had the shortest memory (4.04 ms ± 0.96), lower than both the control (*t* ≈ 8.41, *p* ≈ 1.59 × 10^−16^, Cohen’s *d* = 0.41) and washout (*t* ≈ −19.88, *p* ≈ 4.12 × 10^−73^, Cohen’s *d* = −1.07) conditions. The control condition was in the middle (4.47 ms ± 1.14) and significantly lower than the washout condition (*t* ≈ −8.24, *p* ≈ 5.9 × 10^−16^, Cohen’s *d* = −0.41), which had the longest average memory (4.82 ms ± 0.39).

Collectively, these results indicate that the dynamics induced by DPT are distinct from the drug-free state: The single-neuron activity in the DPT condition is characterized by higher entropy, less reversible dynamics, as well as a shorter “memory” in each neuron (although the total AIS was surprisingly unchanged). These results are broadly consistent with what we might expected based on the EBH.

### Network Measures

After constructing the multivariate transfer entropy network (for details, see the Materials and Methods section), we analyzed the structure of directed, pairwise dependencies between neurons. Friedman’s test found small but significant differences between conditions in the log-transformed total information flowing into each neuron (*Q* ≈ 87.35, *p* ≈ 1.08 × 10^−19^). Post hoc analysis found that, once again, there was no significant difference between the control (−14.79 ± 5.24) and washout (−14.91 ± 4.21) conditions, but that the DPT condition had significantly less mTE (−16.15 ± 3.85) than either control (*t* ≈ 8.53, *p* ≈ 6.04 × 10^−17^, Cohen’s *d* = 0.3) or washout (*t* ≈ −11.46, *p* ≈ 1.91 × 10^−28^, Cohen’s *d* = −0.31) condition.

**Figure 2.**
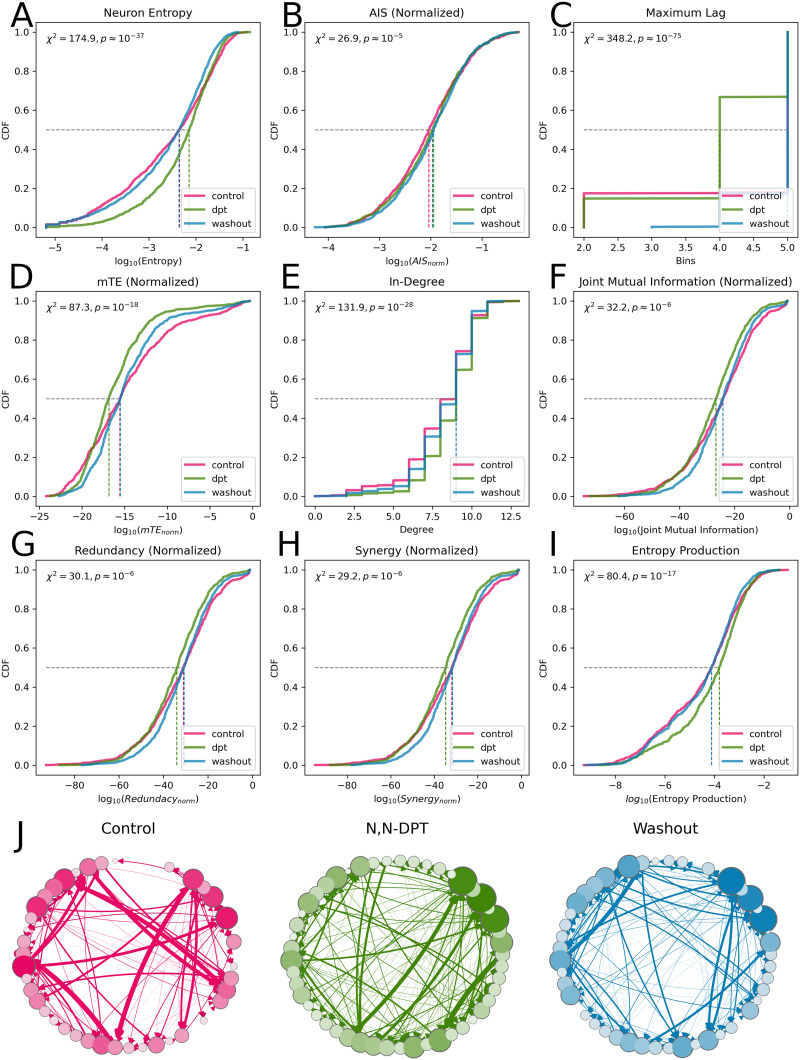
(A) A cumulative distribution function (CDF) plots of the individual neuron entropies for the three conditions (pink: control, green: DPT, blue: washout). The Friedman’s *χ*^2^ statistic is computed from all three distributions. (B–I) CDF plots for the following measures: AIS, AIS maximum lag, total in-coming mTE, in-degree, joint mutual information from two parents onto a single target, redundant information synergistic information, and entropy production. (J) Visualization of a representative mTE network from a single culture during all three conditions. Visual inspection shows that the DPT condition has an increased number of weak (thin) edges when compared with the control condition, consistent with the finding that the in-degree of each neuron has increased even as overall information flow decreases.

Curiously, if we consider the discrete in-degree, rather than considering the total information in-flow, we find the opposite pattern: (*Q* ≈ 131.9, *p* ≈ 2.28 × 10^−29^). There is no significant difference in in-degree between the control (8.08 ± 2.07 edges) and washout (8.27 ± 1.81 edges) conditions; however, the DPT condition has a significantly greater in-degree (8.71 ± 1.6 edges) than both the control condition (*t* ≈ −10.37, *p* ≈ 8.81 × 10^−24^, Cohen’s *d* = −0.34) and the washout condition (*t* ≈ 10.57, *p* ≈ 1.33 × 10^−24^, Cohen’s *d* = 0.25). This is curious as it suggests that, in the DPT condition, there is an increase in low-level connectivity, but that the strength of individual edges is also reduced: a proliferation of weak connections. For visualization of an example network, see Figure 2.

**Figure 3.**
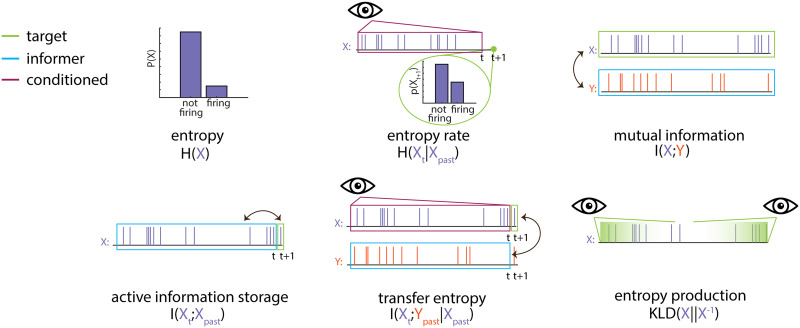
Schematic of information theoretic measures. Six measures are shown on two sample neurons, *X* (purple) and *Y* (orange). For all measures, the target or measured feature is shown outlined in green, variables that inform on the target are shown outlined in blue, and conditional variables are shown outlined in magenta. Note that because mutual information is a symmetric measure, variables outlined in green or blue are interchangeable within a panel. This has been indicated with double-headed arrows in all cases. Conditioned variables are shown with an eye to indicate that the relationship between the targets, and informer variables are dependent on the observed state of the conditioned variable. Please see the technical terms defined throughout the paper Materials and Methods sections for written descriptions of each measure.

This hypothesis is supported by an analysis of local circuit density in the network: commonly called the “clustering coefficient” (Holland & Leinhardt, 1971; Watts & Strogatz, 1998). Briefly, the clustering coefficient gives a measure of local integration: For each neuron, it quantifies how many of that neuron’s neighbors are also neighbors (i.e., form closed triangles). Friedman’s test found significant differences in the log-transformed clustering coefficient between all conditions (*Q* ≈ 211.73, *p* ≈ 1.06 × 10^−46^), and post hoc analysis found significant differences between all pairs of conditions. The control condition had the lowest log-transformed clustering coefficient (−1.91 ± 0.61) compared with DPT (−1.7 ± 0.4, *t* ≈ −15.22, *p* ≈ 2.54 × 10^−46^, Cohen’s *d* = −0.39) and washout (−1.63 ± 0.39, *t* ≈ −8.87, *p* ≈ 4.51 × 10^−18^, Cohen’s *d* = −0.18) conditions, and the washout condition was significantly higher than the DPT condition (*t* ≈ −8.87, *p* ≈ 4.51 × 10^−18^, Cohen’s *d* = −0.18), although note the weak effect size. These results suggest that addition of DPT is associated with an increase in weak, local integration: While the total amount of information coming into each neuron is decreased, more locally clustered weak connections are allowed to open. Curiously, unlike many of the other metrics, this effect persists even after the drug is washed out.

Collectively, these results not only challenge simplistic stories such as “increased connectivity” or “decreased connectivity” but also suggest a more nuanced change in the communicative structure of the network, typified by both an overall decrease in the total information flow and an increase in the number of weak open connections.

### Higher Order Statistical Synergy

When considering higher order information integration (statistical synergy), we found weak but significant patterns consistent with prior results. Friedman’s test on the log-transformed normalized synergy found significant differences between the conditions (*Q* ≈ 29.2, *p* ≈ 4.57 × 10^−7^). Post hoc analysis found no significant difference between the control (−33.21 ± 15.84) and washout (−32.24 ± 13.01) conditions, but a weak, significant decrease in log-transformed synergy in the DPT condition (−35.54 ± 13.64) compared with both control (*t* ≈ 4.35, *p* ≈ 1.51 × 10^−5^, Cohen’s *d* = 0.16) and washout (*t* ≈ −7.43, *p* ≈ 2.73 × 10^−13^, Cohen’s *d* = −0.25) conditions. These results tentatively suggest that, when exposed to DPT, the individual neurons are “integrating” less information from pairs of inputs then they ordinarily would. This finding was unexpected, as previous research has found that loss of synergy is generally associated with decreased conscious awareness (Luppi et al., 2021, 2023), although this prior work has been done exclusively at the whole-brain level. However, we stress that these are tentative results for two reasons: the first is that different redundancy functions or formulations of the partial information decomposition (PID) may return different synergies (Kolchinsky, 2022), and the second is that we only considered the case of two parents and a single target. Higher order combinations may show quantitatively different patterns of information integration, although such an analysis is beyond the scope of this project.

## DISCUSSION

In this paper, we have described how the serotonergic psychedelic N,N-DPT alters the statistics of information dynamics in organotypic cultures before, during, and after drug exposure. We found that concentrations of 10-*μ*M DPT induced a transient dynamic characterized by increased entropy of a single-neuron activity, reduced strong connections between neurons, but simultaneously, a proliferation of weak connections. We found that higher order statistical synergy was decreased, but the temporal irreversibility of neural activity was increased. Collectively, these results paint a complex picture of the effects of DPT on cellular neural circuit dynamics. The decrease in strong connections and reduction in synergistic processing could be described as “disintegration” of the system: In both cases, the smaller proportion of the uncertainty about the future activity of the target neurons can be resolved by learning about other parts of the system. In other words, the behavior of individual neurons could be described as becoming more “autonomous” from the rest of the network: Learning the state of the upstream neurons does not provide as much information about whether the target neuron will “choose” to fire at time *t*. Conversely, however, the increase in in-degree (indicating a growth in weak connections) suggests that this is not the entire story: More channels of information flow may be opening, they are just weaker in nature.

These results are broadly consistent with prior results from whole-brain neuroimaging. The increase in regional entropy is well-documented enough to form the core of the EBH (Carhart-Harris, 2018; Carhart-Harris et al., 2014) (although for a dissenting opinion, see McCulloch et al., 2023). Similarly, the bivariate transfer entropy analysis of MEG data from humans under the influence of LSD and psilocybin found decreased effective connectivity (Barnett, Muthukumaraswamy, Carhart-Harris, & Seth, 2020). To the best of our knowledge, at the time time of writing, there have been no published analyses of how psychedelic drugs impact temporal reversibility or statistical synergy (although Mediano reports that a closely related measure, integrated information, Φ, surprisingly decreases under LSD or psilocybin in a manner somewhat similar to sleep; M. Mediano & Antonio, 2019).

The finding that DPT induces an increase in weak connections may provide insights into the documented ability of tryptamine psychedelics to induce neuroplasticity in neuronal networks. In vitro work has found that exposure to drugs such as LSD and psilocybin produces increased dendritic arborization and synaptogenesis (De La Fuente Revenga et al., 2021; Ly et al., 2018). A naive Hebbian model might suggest that it is the increased information flow between previously disconnected neurons that might drive the emergence of new connections, although we should stress that the transfer entropy network inference algorithm does not claim to recover purely synaptic connections. Future work that can combine spontaneous activity recording with biological analysis of neuroplasticity may be able to explore the connection more directly.

Curiously, despite the consistency with macroscale imaging analyses, the finding that DPT increased the entropy of spontaneous firing activity relative to the control and washout conditions conflicts with two prior cellular-level studies, both of which found that the psychedelic 2, 5-dimethoxy-4-iodoamphetamine (DOI) had an inhibitory effect on spiking activity (Rangel-Barajas, Estrada-Sánchez, Barton, Luedtke, & Rebec, 2017; Wood et al., 2012). One possible explanation for this discrepancy is the different pharmacological profiles of the two drugs: DOI is a substituted amphetamine, while DPT is of the tryptamine class, and they have distinct binding profiles. Another possibility is the difference between in vivo and in vitro studies. Given the overall paucity of research on the effects of psychedelic drugs, further studies will hopefully shed considerable light on these questions.

This study has some limitations that are worth discussing. The most significant is the small absolute number of recordings (11), which makes culture-to-culture comparisons weak (in contrast to neuron- and circuit-level analyses, which are highly powered). The cultures themselves have no behavior or consciousness to speak of, and so the insights that can be gleaned from them about the phenomenological nature of the psychedelic state are limited. The cultures themselves are taken from the dorsal cortex near the somatomotor areas; however, the precise placement of the electrodes varies, which means that there is unavoidable heterogeneity with respect to which neurons are being sampled and what layers are represented. Future replications with larger *N* and, possibly, in behaving animal models will go a long way to addressing these concerns. Recent developments in multilayer imaging from animal cortex (Kajiwara et al., 2021), or machine-learning-based cell type classification (Lee et al., 2021), may augment future studies in this vein. Another significant limitation of this study is that it only focuses on a single drug, N,N-DPT. This means that we cannot be sure to what extent the reported changes to dynamics are specific to DPT or serotonergic psychedelics in general, versus what may be more general effects. Future extensions of this work may explore comparing DPT with other serotonergic psychedelic drugs, as well as nonpsychedelic stimulants (such as cocaine or amphetamine), or atypical psychedelics like ketamine (which targets the glutamatergic neurotransmitter system rather than the serotonergic system). By contrasting different classes of drugs in vitro, future research can more finely detail what changes in neuron-level dynamics correspond to distinct cognitive and behavioral differences induced by psychoactive drugs. Similarly, the use of a single drug gives limited insights into ways that specific receptors might be regulating these dynamical changes. Like other psychedelic tryptamines, DPT binds to the presynaptic 5-HT_1*A*_ receptor in addition to the postsynaptic 5-HT_2*A*_ receptor. These two receptors are believed to have a complex relationship (Carhart-Harris & Nutt, 2017), and further research combining in vitro DPT with coadministration of 2Ar and 1Ar antagonists could provide valuable insights into the way that these receptors regulate information-processing dynamics.

These results should be seen as a first step toward understanding the effects of psychedelics on circuit-level information-processing dynamics. The limitations discussed above suggest natural subsequent studies, including using invasive recordings from behaving animals (where placement of the array can be controlled), studying the dose-response curves with respect to measures like neural entropy, and finally, increasing the population size to improve statistical power. However, despite the limitations, we suggest that this study has provided key insights into the computational effects of psychedelics on mesoscale brain activity.

## CONCLUSIONS

In this study, we showed that the serotonergic psychedelic N,N-DPT disrupts the information-processing dynamics of cortical tissue in in vitro organotypic cultures, with some disruptions appearing to be reversible, while others persist postexposure. The psychedelic increased the entropy of spontaneous neural firing activity while decreasing the temporal reversibility and altered the connectivity patterns of neural circuits, reducing the overall information flow coming into each neuron but increasing the total number of significant connections. These different effects present a nuanced picture, largely irreducible to simple stories of “increasing integration” or “decreasing integration” and instead point to a rich area of future work more carefully characterizing the effects of psychedelics on information-processing, and computational, dynamics in the brain.

## MATERIALS AND METHODS

### Organotypic Culture Preparation, Data Collection, and Preprocessing

Organotypic cultures were prepared according to the methods described in Ito et al. (2011) and Tang et al. (2008). Briefly, we used Sprague-Dawley strain postnatal rats, which were, on average, 5 days old. These animals were approved by the Indiana University Animal Care and Use Committee (IUCAC), and all proper protocols for animal care were followed. The overall procedure involved extracting their brains and slicing them in the coronal plane using a vibrotome to achieve a thickness of 400 *μ*m. After this process, the slices were placed in trays with culture medium in an incubator for a time period between 2 and 4 weeks. The culture medium in the trays was replaced by half every 3 days. The composition of the culture medium is as following: 1 L of minimum essential medium (Sigma-Aldrich), 500 ml of Hank’s balanced salt solution (Sigma-Aldrich), 500 ml of heat inactivated horse serum (Sigma-Aldrich), 2 ml of PSN antibiotic mixture, and 10 ml of L-glutamine.

All animal tissue samples were prepared according to guidelines from the National Institutes of Health, and all animal use procedures were approved by the IUCAC. After 2–4 weeks of maturation, cultures were recorded on a 512-microelectrode array, with 5-*μ*m-diameter electrodes arranged in a triangular lattice with an interelectrode distance of 60 *μ*m (Litke et al., 2004). Data were sampled at a high temporal resolution of 50 *μ*s.

Each culture was recorded for 3 hr. The first hour was the control condition; spontaneous activity was recorded under normal conditions. A “placebo” of empty dimethylsulfoxide (DMSO) vehicle was added to the culture media. Following the control hour, the irrigation system was flushed, and a second batch of culture medium containing 10 *μ*M of N,N-DPT solution in DMSO (Cayman Chemical Company) was introduced. The concentration of 10 *μ*M was chosen to be consistent with prior work on in vitro analysis of psychedelic drugs (Ly et al., 2018). Cultures were recorded from for another hour (the drug condition), before the system was again flushed and the original, drug-free media were reintroduced. Recordings were stopped during media turnover to avoid artifacts.

Following recording, the three 1-hr datasets were appended, and spike-sorting was done using the kilosort3 software package (Pachitariu et al., 2023), in a Python3.7 environment. Following spike-sorting, the resulting rasters were rebinned to 1-ms frames. Rasters were excluded from analysis if they contained less than 30 neurons, resulting in a final count of 11 viable datasets.

### Information Dynamics and Network Inference

Information dynamics is a quantitative framework used to analyze how the elements of a complex system interact and collectively “compute” the future trajectory of a system (J. T. Lizier, 2013). By drawing on analogy with digital computation, the information dynamics framework breaks “computation” in complex systems down into a set of distinct dynamical features, including information storage (analogous to memory, or autocorrelation), information flow or transfer, and information modification or “integration.”

For an element *X* in a stochastic dynamical system, the simplest measure of information structure is the Shannon entropy of that element: How uncertain are we, as observers, about the state *X* will adopt at time *t*? Formally:

$$H\left(X_{t}\right)=-\sum_{x\in 𝒳}P\left(x\right)\log_{2}P\left(x\right)$$ (1)

where 𝒳 is the support set of *X* and *P*(*x*) is the probability of observing that *X* = *x*. However, the Shannon entropy has no temporal component: it assumes that, at every time *t*, the system *X* randomly selects its state according to the probability distribution *P*(*x*).

#### Active information storage.

The simplest measure of information dynamics is the active information storage, which quantifies how much the past state of *X* constrains the possible next state *X*_*t*_ using the mutual information:

$$\begin{array}{l} AIS\left(X\right)=I\left(X_{\text{past}};X_{t}\right)\\ \;=H\left(X_{t}\right)-H\left(X_{t}|X_{\text{past}}\right) \end{array}$$ (2)

where *X*_*past*_ refers to a potentially multidimensional embedding of the past states of *X*.

We can rewrite Equation 2 as a kind of “information regression” that details how information about *X*’s next state is distributed over time (J. Lizier & Rubinov, 2012):

$$H\left(X_{t}\right)=AIS\left(X\right)+H_{\mu }\left(X\right)$$ (3)

Here, *H*_*μ*_(*X*) is the conditional entropy rate *H*(*X*_*t*_∣*X*_*past*_): all that uncertainty about *X*_*t*_ that is not resolved by learning the past of *X*.

For each neuron in each recording, for each condition, we inferred the AIS using a nonuniform embedding algorithm provided by the IDTxl package (Wollstadt et al., 2019). Briefly, the nonuniform embedding procedure iterates through lags 1..*τ*_max_ (inclusive) and tests whether the addition of each subsequent lag significantly increases the AIS, conditional on all previously selected lags, up to some maximal lag *τ*_max_. For more details, see Faes, Nollo, and Porta (2011) and the IDTxl documentation. Here, *τ*_max_ was chosen to be five bins, and 1,000 shuffled nulls were used for null hypothesis significance testing. To control for the effects of variable firing rates, we report the normalized active information storage: *AIS*(*X*)/*H*(*X*_*t*_).

#### Multivariate transfer entropy.

The AIS quantifies how much information the past of a single element discloses about its own future (the amount of information “stored” in *X*). To quantify how much information “flows” from one element to another, we must measure how the past of other elements of the system constrains *X*_*t*_. This is done with the multivariate transfer entropy (Novelli et al., 2019; Schreiber, 2000). For a set of parent elements **Z**, we can quantify how much information the past of **Z** discloses about the next state of *X* with the conditional mutual information:

$$mTE\left(Z\to X\right)=I\left(Z_{\text{past}};X_{t}|X_{\text{past}}\right)$$ (4)

In the context of the information regression, we now have the following:

$$H\left(X_{t}\right)=AIS\left(X\right)+mTE\left(Z\to X\right)+H_{\mu }\left(X\right)$$ (5)

where *H*_*μ*_ is now given by *H*(*X*_*t*_∣*X*_*past*_, **Z**_*past*_). The *mTE* is appealing in that it accounts for potentially higher order synergies between multiple *Z*_*i*_, *X*_*j*_ ∈ **Z**, as well as not double-counting redundancies as the bivariate transfer entropy does (Bossomaier et al., 2016). The full *mTE*(**Z** → *X*) is a multivariate measure, more naturally applicable to hypergraphs than bivariate networks; however, a bivariate network that still accounts for redundancies and synergies can be recovered by defining the weight of each directed edge as *I*(*Y*_*past*_ : *X*_*t*_∣$Z_{\text{past}}^{-Y}$, *X*_*past*_), where **Z**^−*Y*^ refers to the set of all *Z*_*i*_ ∈ **Z** excluding *Y*.

For large systems with finite datasets, it is impossible to account for all possible parents, as well as all possible lags. Here, we used the IDTxl package (Wollstadt et al., 2019) to implement a modified version of the algorithm described in Novelli et al. (2019). IDTxl implements a greedy search, coupled with extensive null hypothesis surrogate testing to infer an optimal parent set **Z** and the embedding for both *X*_*past*_ and **Z**_*past*_; however, the runtimes can still be excessive. The time complexity for a full network inference is *O*(*N*^2^ × *d* × *τ*_*max*_ × *S*), where *N* is the number of neurons in the network, *d* is the eventual average in-degree of each neuron, *τ*_max_ is the maximum search depth, and *S* is the number of surrogates (M. Wibral & Wollstadt, 2021). Given limitations in available computing resources, we first prefiltered the set of prospective parents for each target by removing any neurons that did not have any significant bivariate transfer entropy onto the target over a range of 1–30 bins of lags for the source and 5-ms bins of lag for the target. Significance was tested using the analytic null estimator (Barnett & Bossomaier, 2012) as implemented by the JIDT (Java information dynamics toolkit) (J. T. Lizier, 2014). The results of the analysis were fed into the IDTxl mTE estimator. Following prior work on transfer entropy network inference in neural cultures (Ito et al., 2011; Nigam et al., 2016; Shimono & Beggs, 2015; N. Timme et al., 2014), we constrained the multivariate transfer entropy inference to only consider one bin of source history, fixed by the lag that maximized the significant bivariate transfer entropy. The parent set **Z** for each neuron, in each culture, in each condition was inferred in parallel (requiring approximately 5,000 unique optimizations), and significance testing was done using null distributions of 250 circularly shifted surrogates. The circular shift was chosen to preserve the autocorrelation of each neuron. To control for the effects of variable firing rate, we report the normalized multivariate transfer entropy: *mTE*(*Y* → *X*∣**Z**^−*Y*^)/*H*(*X*_*t*_).

#### Partial information decomposition and synergy.

The final information dynamic we explored is information modification (J. T. Lizier, 2013), sometimes also referred to as information integration. Information modification has been associated with “computation” in neural systems previously (Newman et al., 2022) and refers to novel information generated when a single neuron’s future is constrained by the joint state of multiple inputs simultaneously (J. T. Lizier et al., 2013). Following previous work (Newman et al., 2022; N. M. Timme, Ito, et al., 2016; Varley et al., 2023), we operationalized information modification with the statistical synergy, as computed using the PID framework (Williams & Beer, 2010).

Since its development by Williams and Beer in 2012, the PID framework has been widely applied across a variety of fields, including neuroscience (Luppi et al., 2022; Newman et al., 2022), clinical care research (Luppi et al., 2023), sociology (Varley & Kaminski, 2022), climatology (Goodwell & Kumar, 2017), machine learning (Ehrlich, Schneider, Priesemann, Wibral, & Makkeh, 2023), as well as to philosophical questions such as “emergence” (P. A. M. Mediano et al., 2022; Varley & Hoel, 2022), and consciousness (Luppi et al., 2021). Briefly, the PID provides a scaffold by which the information that multiple sources disclose about a target can be decomposed into nonoverlapping “atomic” components of information. Consider the case where two parent neurons *Y*_1_, *Y*_2_ disclose information about a target neuron *X*. The total information that both parents disclose about the target can be quantified with the joint mutual information: *I*(*Y*_1_, *Y*_2_; *X*); however, this is a lump-sum measure and treats *Y*_1_ and *Y*_2_ as a coarse-grained macrovariable and reveals nothing about how the information about *X* is distributed over the *Y*_*i*_s. The PID solves this issue by decomposing:

$$I\left(Y_{1},Y_{2};X\right)=Red\left(Y_{1},Y_{2};X\right)+Unq\left(Y_{1};X/Y_{2}\right)+Unq\left(Y_{2};X/Y_{1}\right)+Syn\left(Y_{1},Y_{2};X\right)$$ (6)

The term *Red*(*Y*_1_, *Y*_2_; *X*) is the redundant information about *X* that could be learned by learning either the state of *Y*_1_ alone or the state of *Y*_2_ alone. The term *Unq*(*Y*_*i*_; *X*/*Y*_*j*_) is the unique information about *X* that can only be learned by observing *Y*_*i*_. The final term, *Syn*(*Y*_1_, *Y*_2_; *X*) is the synergistic information about *X* that can *only* be learned when both the states of *Y*_1_ and *Y*_2_ are observed simultaneously. We can also decompose the marginal mutual informations:

$$I\left(Y_{1};X\right)=Red\left(Y_{1},Y_{2};X\right)+Unq\left(Y_{1};X/Y_{2}\right)$$ (7)

$$I\left(Y_{2};X\right)=Red\left(Y_{1},Y_{2};X\right)+Unq\left(Y_{2};X/Y_{1}\right)$$ (8)

The result is an underdetermined system of three equations and four unknown values (the redundant, synergistic, and two unique information atoms): If any one term is computed, the remaining three can be solved “for free.” Here, we used the *I*_*BROJA*_ measure of unique information (Bertschinger, Rauh, Olbrich, Jost, & Ay, 2014), as it guarantees a nonnegative decomposition. For each network, in each condition, we computed the bivariate PID for every instance of the two-parent/single-target motif with the BROJA-2PID package (Makkeh, Theis, & Vicente, 2018), as provided by the IDTxl package (Wollstadt et al., 2019). For each parent, we used the same optimal lag as was used in the mTE network inference. To control for variable firing rates, we report the normalized synergy *Syn*(*Y*_1_, *Y*_2_; *X*)/*H*(*X*_*t*_).

#### Reversibility and entropy production.

To assess whether DPT altered the temporal reversibility of cortical activity, we computed the entropy production in the neuron level time series of spiking activity. Based on Lynn et al. (2021) and Roldán and Parrondo (2012), we estimated the entropy production with the Kullback-Leibler divergence between the forward and reverse time spike trains for each neuron: *D*_*KL*_($\vec{X}\|\overset{\leftarrow }{X}$). For a discrete random variable *X* that transitions from state *x*_*i*_ to state *x*_*j*_ according to a stationary transition probability matrix *P*(*x*_*i*_ → *x*_*j*_), the entropy production is given by the following:

$$D_{KL}\left(\vec{X}\|\overset{\leftarrow }{X}\right)≔\sum_{x_{i},x_{j}\in 𝒳}P\left(x_{i}\to x_{j}\right)\log\left(\frac{P\left(x_{i}\to x_{j}\right)}{P\left(x_{j}\to x_{i}\right)}\right)$$ (9)

If *P*(*x*_*i*_ → *x*_*j*_) = *P*(*x*_*j*_ → *x*_*i*_) for all *x*_*i*_, *x*_*j*_, then the system is said to obey “detailed balance” and is at thermodynamic equilibrium: There is no “flow of time” from the perspective of the system. The flow from past to future and from future to past are indistinguishable. On the contrary, if *P*(*x*_*i*_ → *x*_*j*_) ≠ *P*(*x*_*j*_ → *x*_*i*_), then the system has broken detailed balance and is operating far from equilibrium (Lynn et al., 2021).

To ensure that the state spaces were large enough to capture rich temporal dynamics, we used a lossless coarse-graining procedure on each neuron’s spike train: The time series was compressed into nonoverlapping, successive 5-ms bins. Each macroframe could be in 1 of 32 possible states, and we computed the transition probability matrix (TPM) from the sequence of successive macroframes. To satisfy the constraints of the *D*_*KL*_, we only included transitions where both the transition *x*_*i*_ → *x*_*j*_ and *x*_*j*_ → *x*_*i*_ were observed.

#### Clustering coefficient.

The local clustering coefficient (Holland & Leinhardt, 1971; Watts & Strogatz, 1998) for each node in each network was computed using the clustering() function from the NetworkX package (Hagberg, Schult, & Swart, 2008). Briefly, the local clustering coefficient of a node quantifies was proportion of that nodes neighbors are themselves connected. A high value of the coefficient indicates greater local integration.

## ACKNOWLEDGMENTS

T.F.V. and M.P. are supported by the National Science Foundation Research Traineeship (NSF-NRT) Grant 1735095, Interdisciplinary Training in Complex Networks and Systems. M.P. is also supported by the National Science Foundational Graduate Research Fellowships Program (NSF GRFP). J.M.B. is supported by Expeditions: Mind in Vitro–Computing with Living Neurons National Science Foundation 2123781 subcontract to J.M.B. This work was supported by the Source Research Foundation.

## SUPPORTING INFORMATION

Included in the Supporting Information are python scripts used for computing the bivariate TE, the mulitvariate TE, the PID, and the entropy production using IDTxl. Also included are CSV files of the spike times for each culture and each neuron in 1- and 5-ms sampling rates. Supporting information for this article is available at https://doi.org/10.1162/netn_a_00408.

## AUTHOR CONTRIBUTIONS

Thomas F. Varley: Conceptualization; Formal analysis; Funding acquisition; Investigation; Methodology; Visualization; Writing – original draft. Daniel Havert: Software. Leandro Fosque: Data curation; Formal analysis; Writing – review & editing. Abolfazl Alipour: Data curation. Naruepon Weerawongphrom: Data curation. Hiroki Naganobori: Data curation. Lily O’Shea: Data curation. Maria Pope: Visualization. John Beggs: Conceptualization; Supervision; Writing – review & editing.

## FUNDING INFORMATION

Thomas Varley, Source Research Foundation, Award ID: N/A. Thomas Varley, National Science Foundation (https://dx.doi.org/10.13039/100000001), Award ID: 1735095. John Beggs, National Science Foundation (https://dx.doi.org/10.13039/100000001), Award ID: 2123781. Maria Pope, National Science Foundation (https://dx.doi.org/10.13039/100000001), Award ID: 1735095.

## Supplementary Material



## TECHNICAL TERMS

Out-degree: The number of out-going edges from a node in a network.
Entropy: A measure of uncertainty about the outcome of a random draw from a distribution. Formally: *H*(*X*) = −∑_*x*∈𝒳_ *P*(*x*) log *P*(*x*), where 𝒳 is the support set of *X*.
Active information storage: For a temporally extend process *X*, the AIS is the information about the state of *X*_*t*_ at time *t* disclosed by the past. Formally, *AIS*(*X*_*t*_) = *I*(*X*_*past*_; *X*_*t*_).
Entropy production: A measure of the time reversibility of a temporal process. Formally, *D*_*KL*_($\vec{X}\|\overset{\leftarrow }{X}$).
Multivariate transfer entropy: The information that the past of one variable **X** discloses about the next state of a target variable *Y* conditioned on the past of all other parents of *Y*. Formally, *I*(*X*_*past*_; *Y*_*t*_∣*Y*_*paste*_, **Z**_*past*_, where **Z** is the set of all parents of *Y* excluding *X*.
Transfer entropy: The information that the past of one variable *X* discloses about the next state of a target variable *Y*, conditioned on *Y*’s own past. Formally, *TE*(*X* → *Y*) = *I*(*X*_*past*_; *Y*_*t*_∣*Y*_*past*_).
Local clustering coefficient: A measure of how many triangles a given node participates in relative to the total number of triangles it could possibly participate in given its degree.
In-degree: The number of in-coming edges to a node in a network.
Maximum lag: The maximum distance in the past accounted for when computing the AIS.
Partial information decomposition: A technique for decomposing the information that two sources (*X*_1_ and *X*_2_) disclose about a single target *Y* into redundant, unique, and synergistic components.
Support set: For a random variable *X*, the support set of *X*, denoted as 𝒳, is the set of all possible states *X* can adopt.
Mutual information: The amount of uncertainty about a variable *X* reduced upon learning the state of some other variable, *Y*. Formally, *I*(*X*; *Y*) = *H*(*X*) − *H*(*X*∣*Y*), where *H*(*X*∣*Y*) is the conditional entropy of *X* given *Y*.
Redundant information: The information about a target *Y* that could be learned by observing either *X*_1_ alone or *X*_2_ alone.
Unique information: The information about a target *Y* that can only be learned by observing *X*_*i*_.
Synergistic information: The information about a target *Y* that can only be learned by observing the joint state of *X*_1_ and *X*_2_ simultaneously.
Kullback-Leibler divergence: The information gained when updating one’s belief from a prior distribution to a posterior distribution. Formally, *D*_*KL*_(*P*‖*Q*) = −∑_*x*∈𝒳_ *P*(*x*) log *P*(*x*)/*Q*(*x*), where *P* and *Q* are both probability distributions on the support set 𝒳.
